# Research Culture and Integrity in Japan: A Qualitative Study

**DOI:** 10.1007/s11948-026-00591-2

**Published:** 2026-03-14

**Authors:** Tatsuya Ito, Mika Suzuki, Keiko Sato, Sayaka Takenouchi, Satoshi Kodama, Tetsuji Iseda

**Affiliations:** 1 Regulatory Science and Pharmaceutical Informatics, School of Pharmaceutical Sciences, Wakayama Medical University, Wakayama, Japan; 2 Kyoto College of Medical Science, Kyoto, Japan; 3 University Research Board, The University of Osaka, Osaka, Japan; 4 Research Center on Ethical, Legal and Social Issues, The University of Osaka, Osaka, Japan; 5 Health Informatics, School of Public Health, Graduate School of Medicine, Kyoto University, Kyoto, Japan; 6 Human Health Sciences, Graduate School of Medicine, Kyoto University, Kyoto, Japan; 7 Division of Philosophy, Graduate School of Letters, Kyoto University, Kyoto, Japan; 8 Division of Contemporary Culture, Graduate School of Letters, Kyoto University, Kyoto, Japan

**Keywords:** Research integrity, Research misconduct, Focus group interview, Research culture

## Abstract

There have been numerous instances of research and funding fraud by Japanese researchers that have discredited Japanese research, and thus ministries, universities and other institutions have attempted to prevent research misconduct and promote research integrity through measures such as policy amendments and education. However, it is unclear whether those measures have been effective, because research misconduct continues to occur. In this study, we conducted focus group interviews with 25 participants, including both researchers and administrators, from various academic disciplines. The interviews were guided by seven semi-structured questions based on the Promoting Integrity as an Integral Dimension of Excellence in Research protocol. Our analysis of the resulting data identified 20 themes. Regarding research integrity, participants highlighted boundary ambiguity and the need for a new definition. In relation to the research culture, the following barriers were identified: the laboratory environment, job security for junior researchers and middle-senior researchers, and pressures faced by principal investigators. In terms of policies, participants observed that research integrity regulations were not widely disseminated, and proposed several methods to provide education, including informational materials. Regarding improvements to the current environment, participants mentioned performance evaluation, an open research environment, a supportive research environment, and sharing of previous cases. While numerous issues were raised, several solutions were also proposed. We found that gaining access to the collective knowledge of faculty, students, and administrators is the best way to foster a strong research culture and develop research integrity.

**Supplementary Information:**

The online version contains supplementary material available at 10.1007/s11948-026-00591-2.

## Background

In Japan, there have been numerous cases of misconduct and research funding fraud by academics that have impugned Japan’s research credentials (Kupferschmidt, 2018; Else, 2019). This has resulted in not only a decline in research quality, but also a shortage of qualified researchers and wasted research funds, initiating a negative spiral that has resulted in significant damage to the research environment and research education systems. This misconduct has also had a negative impact on the public to which research results are reported. In response, government ministries, universities, and other institutions have implemented various efforts aimed at preventing research misconduct and promoting research integrity (e.g., amending relevant policies). The Code of Conduct for Scientists was issued by the Science Council of Japan in 2006 and revised in 2013, while Guidelines for Responding to Misconduct in Research Activities were issued by The Ministry of Education, Culture, Sports, Science and Technology in 2006 and revised in 2014. In addition, the Japan Science and Technology Agency has established a research integrity portal on its website (https://www.jst.go.jp/kousei_p/en/index.html) that provides guidelines, overseas trends, e-learning content, and other information. In addition, the Japan Agency for Medical Research and Development provides research ethics education programs, research integrity networks, and research projects related to research integrity (https://www.amed.go.jp/en/kenkyu_kousei/index.html). The Association for the Promotion of Research Integrity was established in April 2016 to provide support for research activities, focusing on research integrity consultation to universities and research institutions (https://www.aprin.or.jp/en).

Research misconduct is a global problem, and thus efforts to address it are underway in various countries and regions. In the United States, the Office of Research Integrity is the regulatory authority that is attempting to crack down on research misconduct. Meanwhile, regional and national codes of practice have been established in Europe and Asia, and various independent organizations are engaged in research integrity promotion and research misconduct consultation. An example of a sound comprehensive approach is an organization initiated by the European Union, Promoting Integrity as an Integral Dimension of Excellence in Research (PRINTEGER, https://printeger.eu/). PRINTEGER aims to increase research integrity by promoting an enhanced research culture rather than a passive management system imposed from outside the research community. PRINTEGER conducted a study in four countries using focus group interviews to explore issues related to research integrity, and found that pressure to publish results led to research misconduct, and that changes in research evaluation methods, such as amending reward systems and incentives to promote research integrity, and changes in research culture, such as the active promotion of a culture of research integrity by research leaders, were key responses. The study identified the factors that cause researchers to engage in research misconduct and proposed several solutions. These are common issues facing researchers and research institutions worldwide, and thus PRINTEGER’s approach is aimed at creating a research culture that is free of the need to engage in research misconduct. Therefore, their methods are useful in relation to rethinking the research culture that exists in Japan.

Japanese universities and research institutions have begun to implement various measures aimed at preventing research misconduct, as well as providing education on research integrity. However, research misconduct continues to occur, even though the guidelines have been revised. To improve research behavior, it is not sufficient to simply implement policies and guidelines. It is also necessary to investigate laboratory practices in an effort to identify the factors that lead to misconduct. Even though such investigation is essential for developing appropriate countermeasures, investigations into individual cases are usually conducted privately, and the findings are not made publicly available. An alternative, albeit somewhat indirect approach, is a survey of the perceptions of researchers and research-related personnel in relation to research integrity, which can provide valuable information. However, this type of investigation is scarce, and has rarely been conducted in Japan.

Therefore, in this study, we adopted a focus-group approach based on the PRINTEGER research protocol with the aim of understanding the conditions and background in which research misconduct can occur, analyzing the relevant cultural factors, summarizing the issues, and proposing solutions based on the research culture, regulatory complexity, and researcher evaluation that exist in Japan.

## Methods and Materials

### Protocol

Focus group interviews using seven semi-structured questions were conducted based on the protocol adopted by the PRINTEGER project, which was translated into Japanese.

### Participants

We interviewed five focus groups containing a total of 25 participants across various disciplinary fields (see Table 1). The participants were researchers, faculty members, and research administrators affiliated with Kyoto University in positions of varying authority and responsibility. Kyoto University, founded in 1897 as Japan’s second oldest university, ranked 61st in the Times Higher Education World University Rankings 2026, making it the second-highest ranked research-intensive university in Japan. Three groups consisted of academic researchers, while the other two groups consisted of university staff in charge of research management and support. Instead of using random sampling, we invited people who had been identified as appropriate subjects to participate. This did not result in a statistically representative sample, but this is not always feasible in a small, exploratory, qualitative study. Consideration was given to ensuring that there was no gender bias in the groups.

**Table 1:** Focus group participant information

	Group 1	Group 2	Group 3	Group 4	Group 5
Subject number	5 (M3:F2)	5 (M3:F2)	6 (M4:F2)	3 (M2:F1)	6 (M5:F1)
Group composition	Junior researchers: Doctoral students	Middle-senior researchers: Assistant professors	Senior researchers: Professors and principal investigators	Research managers: Deans of faculty and institution directors	Research administrators: University administrators and specialists
Disciplinary field	Biomedical science, Humanities, Natural science	Biomedical science, Humanities, Natural science	Humanities, Medical science, Natural science	Engineering, Natural science, Humanities	Natural science, Humanities

M: Male, F: Female

Initially, we made enquiries around Kyoto University in an effort to identify suitable subjects for our study, and then sent an email to the selected candidates explaining the study. We sent a follow-up email two weeks later to candidates who had not yet responded, and if the candidate had still not responded a week later, an alternative candidate was identified and the same steps were repeated. We obtained information on all candidates who had agreed to participate in the study and assigned them to an appropriate focus group. Then, we contacted the participants individually to identify convenient times for the focus group interviews. After the focus group interview schedule was determined, an email was sent to the participants advising the date, time, location, and instructions.

### Ethical Approval

This study was conducted in accordance with the Ethical Guidelines for Medical Research Involving Human Subjects in 2019, subsequently integrated into the Ethical Guidelines for Medical and Biological Research Involving Human Subjects in March 2021. Prior to data collection, approval for this study (R1788) was obtained from the Medical Ethics Committee of the Graduate School of Medicine, Faculty of Medicine, and affiliated hospitals of Kyoto University.

### Procedure

The focus group interviews were conducted from August 2019 to March 2020, with each interview lasting for approximately two hours. All participants were native Japanese speakers, and thus the interviews were conducted in Japanese. The interviews were based on the protocol used in the PRINTEGER project (Kennedy et al., 2018). Before the interviews commenced, the moderator and participants introduced themselves and the moderator outlined the rules to be applied during the session. The main discussion in all of the focus groups was guided by seven semi-structured questions. There were some differences in the questions presented to the researcher participants (Groups 1–3, see Table 2) and those presented to the research managers and support staff (Groups 4 and 5, see Table 3) to reflect their different roles. The questioning method proposed by Krueger and Casey (2015) was used, that is, a warm-up question to introduce the participants, followed by increasingly specific questions targeting the key areas of interest for the study, and ending with a closing question to summarize the session. Data from the interviews were collected in two ways. First, the main data for analysis were collected using digital audio-recorders. Second, a note-taker sat apart from the participants to observe each session while making some basic fieldnotes (Finch & Lewis, 2003).

**Table 2:** Semi-structured questions for Groups 1–3

	Question
Q1: Background (Warm up)	Please tell us your name and what you most like to do when you are not working on research. 1. Can anyone tell me why you conduct research, and what your motivations are? 2. What makes good research?
Q2: Defining bad research	Can anyone tell me what sort of things you consider to be bad research? For example, what sorts of things do you consider to be misconduct or poor practice in research?
Q3: Defining research integrity and what it means in practice for researchers	How would you define research integrity?
Q4: Research culture: expectations and management of research integrity	Can anyone tell me what barriers or challenges you think researchers face in their everyday work that can affect research integrity or misconduct?
Q5: Knowledge, education, and the impact of policies about research integrity on practice	Thinking about policies at your institution (Kyoto University) that relate to research integrity and misconduct, or wider policies such as the Singapore statement (WCRI, 2010) or other national policies that have been developed, can anyone tell me how these impact your research?
SQ6: Support, interpretation, and translation of research integrity policies	Can anyone tell me how you have learnt about standards of good research conduct and integrity?
Q7: What works and what needs improvement	1. Can anyone tell me what you think researchers need to foster and maintain research integrity? 2. Can anyone tell me what you think are the key barriers to the development of research integrity?

**Table 3:** Semi-structured questions for Groups 4 and 5

Q1: Background (Warm up)	In your work, all of you help to support or manage academic research. Can anyone tell me what this work involves?
Q2: Defining bad research	Can anyone tell me what sorts of things you consider to be misconduct or poor practice in research?
Q3: Defining research integrity and what it means in practice for research governance advisors/managers	How would you define research integrity?
Q4: Research culture: expectations and management of research integrity	Could anyone tell me what barriers or challenges you think researchers face in their everyday work that might affect research integrity or misconduct?
Q5: Knowledge, education, and impact of policies about research integrity on practice	Thinking about policies at your institution (Kyoto University) that relate to research integrity, can anyone tell me what sorts of things are included and why they are important?
Q6: Support, interpretation, and translation of research integrity policies	Do you think that the policies developed to address research integrity and misconduct are effective in everyday practice?
Q7: What works and what needs improvement	1. What do you think currently works well in fostering and maintaining research integrity at your institution? 2. Does anyone have any ideas about ways in which your research institution/organization might further develop its integrity policies and reduce misconduct?

### Analysis Methods

We applied a systematic framework approach to identify inductive themes in the focus group transcripts and categorized the results using classic analysis strategy (Krueger & Casey, 2015). All analyses were conducted in Japanese. We read through each transcript to familiarize ourselves with the material, and then created a matrix for each question. Next, we systematically examined and compared the participants’ responses in each transcript in relation to each question. Once all of the transcripts had been categorized based on the responses to each question and initial codes had been assigned, we analyzed and refined the codes, looked for relationships between codes, and either combined similar codes or divided a code into subcodes, continuing this process until the coding framework captured all of the themes that had been identified in the data. Once the coding was finalized, we wrote a description explaining what the coding represented. Finally, we confirmed the description with the participants and then analyzed the data to enable us to prepare a list of themes, subthemes, and a descriptive summary. We noted any differences/similarities within the three groups of academic researchers with different fields of expertise and positions, and the two groups of university staff in charge of research management and research support. The results of our analysis were then translated from Japanese into English.

We illustrate our coding procedure using a concrete example from our study. In Q4 (Research culture: expectations and management of research integrity), a doctoral student in Group 1 said that “The only person who can judge the good things or not is probably the boss in the laboratory. But it is difficult to know if the boss is a trustworthy person or not.” From this response, we assigned *convenient decisions only within the group* as an initial code. In combination with other codes, we refined this to a final code of *concerns about laboratory decisions include convenient decisions only within the group and the reliability of the person presiding over the laboratory*. Then, we summarized the codes into *judgment of misconduct in the laboratory lacks any judgment index in case of difficulty*,* and is undertaken at the convenience of the group*,* in an environment in which everything depends on the credibility of the laboratory leader*. We labeled *issues related to the final decision* as a subtheme. Finally, we summarized all of the subthemes with the relevant phrase to obtain the theme of *Laboratory climate and educational context*.

## Results

We analyzed the responses of the 25 participants in the five focus groups to each question into themes, subthemes, and descriptive summaries, obtaining details of specific problems and suggested countermeasures. This resulted in a total of 20 themes.

### Motivations for Undertaking Research

The themes extracted from each of the three researcher groups are shown in Table 4. All three groups had a positive perception of research, such as contributing to the exploration of intellectual curiosity.

**Table 4:** Themes extracted from responses to Q1-1: Can anyone tell me why you conduct research, and what your motivations are? (Groups 1–3)

Theme	Subtheme
Pursuit of knowledge as a researcher	Interest in unresolved issues
	Contribution to the exploration of intellectual curiosity
	Contribution of research results to society
	The research position as a profession
	Occupation as a secondary choice

I simply want to know what people don’t know before they know it. Middle-senior researcher

### Defining Good Research

Of the three researcher groups, only the group of junior researchers mentioned conducting research using an appropriate methodology (see Table 5).

**Table 5:** Themes extracted from responses to Q1-2: What makes good research? (Groups 1–3)

Theme	Subtheme
Scientifically sound research addressing societal challenges	Conducting research using the correct methodology

I believe that well-designed research, well-designed methodology, and introduction from past research are the key to meaningful results. Junior researcher

### Defining Bad Research, Poor Practices, and Misconduct

Three themes regarding the definition of bad research are presented in Table 6. The responses varied, but included a wide range of questionable research practices.

**Table 6:** Themes extracted from responses to Q2: Defining bad research (Groups 1–5)

Theme	Subtheme
Poor quality research and inadequate methodology	Poor research practices
	Plagiarism among students
	Lack of qualifications and competence
	Inducing results for convenience
	Lack of responsibility for research
	Poor-quality papers
	Lack of research management
	Issues in clinical research
	Lack of consideration for subjects
Pernicious authorship and citation	Customary authorship
	Coercion from journal referees
	Questionable authorship
Dishonesty toward society and the research community	Sanctions against researchers
	Distrust of society

We receive many low-quality papers, which provide insufficient data from which to draw a conclusion. The papers are either too long or make it impossible to draw a conclusion from the data presented. They are basically bad in that they do not make a proper effort to draw a conclusion, such as failing to analyze the literature, or depending heavily on diagrams or photos to reach a conclusion without enough explanation, etc. Senior researcher

In addition, questionable research practices that were mentioned included pernicious authorship and citation. In terms of disloyalty, participants cited dishonesty toward society and the research community.

I wonder if it is bad research when studies convey the message that, for example, if you eat this, you will live longer, because this suggests that a statistically significant difference has been found. Middle-senior researcher

### Defining Research Integrity

Three themes related to defining research integrity are shown in Table 7. Participants indicated responsible research behavior and honesty. Before focusing on the definition of research integrity, participants focused on the line between good and bad research, stating that it was sometimes difficult to determine the level of research integrity because of boundary ambiguity. In addition, the diversity of research areas and ambiguity regarding what constitutes research integrity led managers and administrators to suggest the need for a new definition.

**Table 7:** Themes extracted from responses to Q3: Defining research integrity and what it means in practice for researchers (Groups 1–3) or for research governance advisors/managers (Groups 4 and 5)

Theme	Subtheme
Conceptual ambiguity and definitional gaps	Ambiguity as a result of the diverse range of research fields
	Borderline concerns
	Difficulties of interpretation as the opposite of research integrity
	Need for boundaries for research misconduct
	Definition of research integrity in every research area
	Amendment of research integrity
Responsible research behavior and honesty	Adherence to research rules
	General attitude as a researcher
	Basic concept of research
	Qualities and attitudes as a researcher
Relationship building between the public and researchers	Gap between society’s needs and research goals
	Accountability to society
	Gap in thinking between society and researchers
	Expectations from society

When we talk about research integrity, I think it is something that cannot be determined without some kind of norm, such as this is good and this is bad. I think such norms differ depending on the field. Therefore, I think it is very difficult to define what research integrity is in general. Junior researcher

I guess different fields have different rules and regulations for journal submissions. Therefore, communication between fields is probably important. The era or culture is also important. Research manager

Some groups mentioned relationship-building between the public and researchers.

Can researchers confidently explain the results of their research to society? I guess publishing the research results helps to ensure research integrity by making them accessible to the public. Research administrator

### Barriers or Challenges to Research Integrity

Five themes related to research culture are presented in Table 8. Both young and senior researchers identified numerous barriers and challenges in relation to research evaluation, the research environment, and differences among various research areas. Junior and middle-senior researchers often encountered barriers in the laboratory environment.

**Table 8:** Themes extracted from responses to Q4: Research culture: expectations and management of research integrity (Groups 1–5)

Theme	Subtheme
Laboratory climate and educational context	Education through apprenticeship
	Issues related to the final decision
	Environmental issues considered by junior researchers
	Obstacles regarding researchers’ attitudes within the research community
	Research guidance methods in the laboratory
	Closed laboratory management
	Community rules in the laboratory
	Obstacles that we cannot control ourselves
	The creator and the user of the guidance are different
	Environment in which researchers feel cramped
	Rules and communication challenges in the laboratory
	Environment around improper research
	Background and factors leading to research misconduct
	Inadequate educational environment
	Limitations on research administrators
Positional issues for young researchers	Unstable positions
	Vulnerability of the younger generation
	Identity security and time-limited employment
	Barriers to conducting research for the younger generation
	Evaluation of young researchers based on published papers
Challenges in conducting research for principal investigators	Challenges of running a laboratory
	Conflicts of interest faced by researchers
	Conflicts of interest with external partners
	Differences in positions among organizations and individuals
	Disparities between universities
	External factors for researchers
Challenges regarding reliability of research	Differences between the natural sciences and the humanities
	Difficulty of interpretation in the humanities
	Difficulty in publishing
	Complexity of fraudulent decisions by the research field or organization
	Reliability assurance of research results
	Issues related to research involving human subjects
	Limitations of research quality assurance
Confusion over research rules	Flexibility regarding rule changes
	Difficulties in research guidance in science areas
	Lack of understanding or questions about research integrity
	Limitations of university policies
	Increased burden of procedures
	Policy transformation at the national level

What the community boss says is absolute, and their sense of ethics definitely shapes the atmosphere. Junior researcher.

There are measurement methods that can only be performed in that lab for basic research that were established a long time ago and are considered confidential. There is basically no opportunity for outsiders to judge their validity, and those inside the lab simply assume that they are valid. Middle-senior researcher

Meanwhile, research managers and administrators noted the importance of the educational environment for maintaining quality of research.

Some researchers have reached a certain standard of integrity, while others have not. To compensate for this, institutions provide education, but there are those who learn from that education and those who do not. I always wonder why it doesn’t penetrate in every case. Research administrator

Positional issues regarding junior researchers and middle-senior researchers were also identified.

The position of researchers is weak and unstable. There are few stable positions available to researchers in Japan today. It is a barrier. Junior researcher

Senior researchers identified challenges to conducting research for principal investigators.

There is excessive competition for both research funding and positions, and if they don’t perform during that time, they don’t get another job. Senior researcher

Research managers noted the difficulty faced by humanities researchers in demonstrating novelty

My research sometimes finds relevance in everyday life. When I present the results, I get a lot of comments from people saying ‘I can imagine, that’s something I’ve thought about, too. Research manager.

### Knowledge and Impact of Research Integrity Policies

Two themes related to research integrity policies are presented in Table 9. Most participants knew of the existence of such policies but were currently conducting research without fully understanding all of the policies and regulations, although this had no effect on their research. Research administrators suggested that it is necessary to build a system to educate the researchers about the factors involved in research misconduct. Meanwhile, other groups mentioned the need to provide increased education regarding research integrity.

**Table 9:** Themes extracted from responses to Q5: Knowledge, education, and the impact of policies regarding research integrity on practice (Groups 1–5)

Theme	Subtheme
Policy limitations and the burden of learning research integrity	No effect on research despite lacking knowledge of research integrity
	Differences in educational initiatives between other countries and Japan
	Burden of acquiring research integrity
	Burden of student education
	Increased burden as a result of learning and enforcing research rules
	Limitations of policies to prevent misconduct
Ethics education and researcher protection	Efficient guidance
	How to study the research rules
	Clarification of rules for education
	System building for researcher protection

Rules should be followed, but we have a class in which we think about why such rules exist, and we discuss this in groups so that everyone can think twice about the rules. In our lab, most of the research cases cannot be categorized under the existing rules, and thus I hope that we can use that as an opportunity to have the students think about the policy. Research manager

### Research Integrity Education and Support

One theme in relation to research integrity education and support is presented in Table 10. Participants requested ethics education and mitigating regulatory dissemination barriers.

**Table 10:** Themes extracted from responses to Q6: Support, interpretation, and translation of research integrity policies (Groups 1–5)

Theme	Subtheme
Enhancing ethics education and mitigating regulatory dissemination barriers	Overseas education for research integrity
	Requests for a research integrity educational environment
	Study period on research integrity
	Enhancement of research integrity educational environment
	Creation of a system of regulations and ethics education materials
	Confusion and trial-and-error when learning
	Limitation of learning and use of punishment

The researcher is not aware of the parties involved and probably needs to be alerted to that, and then I wonder if any further monitoring would be too costly, so I’m still thinking that strict punishment for wrongdoing would be more effective as a deterrent. Research administrator

### Promoting Research Integrity and Researcher Needs

Four themes in relation to research integrity and researcher needs are presented in Table 11. Participants demanded improvement in performance evaluation, including the need to create alternative methods of performance evaluation, and measures that they could immediately implement themselves. Some participants had experienced transparency and integrity in the research environment in which middle-senior researchers could interact with people beyond their own research field.

**Table 11:** Themes extracted from responses to Q7: What works and what needs improvement? (Groups 1–5)

Theme	Subtheme
Performance evaluation	Need to create alternative methods of performance evaluation
	Measures that we can immediately implement ourselves
Promoting transparency and integrity in research environments	Promoting research integrity
	Interaction among young researchers
	Measures that require cooperation from the university
	Fostering ethical consciousness
	Reinforcement of research review system
	Curriculum development for research integrity
	Improvement in the preparation of papers
	Measures to prevent research misconduct
Promoting researcher well-being through supportive partnerships	Measures to actively encourage communication
	Measures to deal with the consultation system
	Measures when conducting research
	Measures to provide guidance for students
	New environment for research funding
	Building good relationships with research supporters
Sharing of past cases	Measures from case studies in the past
	Education using bad examples
	Sharing post-disciplinary trends
	Sharing information on research misconduct cases
	Utilization as educational materials through the sharing of research misconduct cases

Our rule in the graduate school was to mix fields and grades, and we had to share a room with people who were not in the same lab or the same grade. Then, a faculty member would approach a student in a different research field and ask, ‘What’s going on with this?’ I was able to hear about the field in which they were currently working. Middle-senior researcher

Senior researchers also suggested the need for researcher well-being through supportive partnerships, such as measures to actively encourage communication.

In my graduate school, there is a system called ‘sub-supervisor,’ in which two students are chosen from completely unrelated areas of the graduate school, and they go to each other once every six months to report on their research. If there is a problem that cannot be discussed in the lab, the student can talk to his/her sub-supervisor. Senior researcher

In terms of sharing past cases, some participants noted the importance of educational information.

Punishment alone is not sufficient for research misconduct. Thus, we not only educate people regarding the policy, but also take cases of research misconduct and turn them into teaching materials. Research administrator

Tables 4, 5, 6, 7, 8, 9, 10 and 11 present the detailed results of the study, which are also provided in the Supplementary Information to facilitate transparency and reproducibility.

## Discussion

The aim of this study was to fully understand the conditions and culture in which research misconduct can occur, summarize the issues, and consider solutions based on the research culture and other conditions in Japan. This is the first study to conduct focus group interviews on research integrity targeting Japanese researchers. We identified 20 themes, and comparisons between groups yielded interesting results from the participants’ perspectives regarding issues and solutions. It was found that the laboratory unit can create a closed environment and fixed relationships, making it difficult for new information regarding research integrity to enter from the outside, and that research itself is a special environment that differs from the public sphere. These findings can be summarized as follows.

### Relationships Between Researchers and Organizations: A Lack of Skills Among Researchers and a Closed Environment that Obstructs Accountability and Reform

Poor-quality papers were considered to be evidence of bad research, especially when there was a lack of experimental reproducibility, self-interpretation of experimental data, insufficient citations of previous studies, conveniently cited references, and insufficient data to enable valid conclusions to be drawn. Although a lack of research skills is a rudimentary issue that is usually corrected within the laboratory’s educational system, it was noted that educational guidance in the laboratory might be inadequate. In general, a laboratory is a small, closed environment that functions under its own rules and approach to information management. To become a researcher, young students, especially in the natural sciences, must join a laboratory and adhere to the numerous rules and guidance provided by the leader, as well as any university policies. Junior and middle-senior researchers in the natural and life sciences have pointed out that research laboratories in Japan often operate in a closed environment, where members are bound by internal rules specific to each laboratory. This insularity is considered to be one of the factors that might contribute to the overlooking of research misconduct within laboratories. It was demonstrated that ethical decision-making is highly contingent on situational factors, emphasizing the influence of context on individual moral judgment and behavior (Jones, 1991). However, the relationship between individuals and organizations in Japan differs from that in Western societies. A hierarchical culture has existed in Japan since ancient times, whereby people avoid expressing their opinions directly to their superiors, sometimes using imprecise or ambiguous language in an effort to avoid confrontation (Iwai & Iwai, 2013; Moeran, 1984; Teichler, 2019). Because principal investigators are competitive in terms of their research activities and sensitive regarding their handling of research information, they often strongly impose their ideas on laboratory staff. Thus, it is difficult for staff to act if they suspect that their supervisors are engaging in research misconduct. This can result in poor research and poor-quality papers if the work is done without careful guidance and adequate review. These laboratories are often an environment lacking in adequate supervision and open discussion. This cultural background might be one reason for the closed environment in which research misconduct tends to occur. It was suggested that the current publication culture leads to questionable research practices among junior and senior researchers in the biomedical science area (Tijdink et al., 2016).

One junior researcher recounted a positive experience whereby although graduate students were assigned to a laboratory and conducted experiments within that laboratory, their desks were located in the graduate students’ living areas, enabling graduate students from various disciplines and laboratories to interact with each other and exchange information freely. By facilitating open discussions among researchers in various research fields during graduate teaching days, it might be possible to encourage the development of a more open environment when these researchers become principal investigators. The establishment of a room that enables graduate students from different research fields to network is an easily implementable strategy.

Some senior researchers suggested that student guidance could be improved using the secondary advisor system, given that it is difficult for junior researchers to talk directly with faculty and other researchers. These human relationships are thought to be the key to avoiding research misconduct, and there was general agreement that the closed laboratory environment should be improved. Building a strong research integrity culture in the organization creates a good environment with shared values and norms, as noted in the Bonn PRINTEGER Statement (Forsberg et al., 2018). The importance of creating an environment that improves the supervisor’s ability to provide support and discuss junior researchers’ expectations was seen as the key to promoting research integrity (Haven et al., 2020; Sørensen et al., 2021).

### Fostering a Culture of Research Integrity: Gaps Between Theory and Practice

While numerous regulations and guidelines are issued by the government, universities, and research institutes aimed at promoting compliance and preventing misconduct, the study participants did not regard them as effective. Many researchers, managers, and administrators felt that it was difficult to keep up with the flow of new regulations and other information. One issue might be that research regulations have their limitations in terms of covering all research areas, and thus research is often conducted without a full understanding of the contents of the regulations. Researchers are required to comply with the regulations that apply in relation to their own fields, but found it difficult to describe the specific actions that they were required to take when conducting research. This can lead to ambiguity regarding what constitutes misconduct, with decisions left to the researchers. Regulations have become a formality because researchers have not been sufficiently informed regarding what they need to do to maintain research integrity.

It was found that researchers had little opportunity to gain a thorough understanding of research regulations while they were undergraduate students, and often learned their research methods from their mentors and university staff. Junior researchers and middle-senior researchers recognized that good research starts with the application of sound methods, and thus it is important to provide training in appropriate research methods and attitudes at the commencement of a research project. Research integrity training should commence at the undergraduate level (Satalkar & Shaw, 2019). One research manager taught a class that made students think deeply about the rules. Furthermore, it is important to acquire the ability to think for oneself using case studies. It was also noted that it is important to not only provide ethics education, but also to consider how to respond to problematic situations (Katsarov et al., 2022).

Senior researchers are responsible not only for research and education, but also for university administration and academic activities outside the university, and have little time to absorb new or revised regulations or to learn new research methods using new technology. It was noted that time available for research was reduced by the need to engage in other tasks (Haven et al., 2020). Achieving a suitable balance among research, education, and administration has long been a problem for senior researchers, who proposed the establishment of a consulting office to proactively disseminate research integrity measures and address the concerns of researchers. As for education regarding research rules and regulations, one research manager recounted an experience whereby faculty and students worked together to create simple educational materials related to research rules and regulations in every research field based on previous cases. This approach to creating researcher-friendly guidance would help researchers to learn about not only the relevant research rules and regulations but also the correct research approach.

The research managers and administrators noted that the most effective deterrent against research misconduct was strict punishment. Additionally, they suggested that sharing research misconduct cases and penalties might help researchers to understand their responsibilities. While they emphasized deterrence through strict punishment and the sharing of misconduct cases, it might be worth considering a greater focus on establishing systems that facilitate the detection of research misconduct and reduce the likelihood of its occurrence, particularly in view of inadvertent violations and ethically ambiguous behaviors. In addition, sharing and reinforcing the value of research integrity within the academic community is also considered important.

### System Design to Promote Research Integrity: Difficulties Regarding Researcher Evaluation

The current approach to researcher evaluation, which is based on research achievements such as published papers and acquisition of research funds, might lead to misconduct and employment instability among junior researchers and middle-senior researchers.

The focus group interviews revealed that each level of Japanese researchers is under considerable pressure. Principal investigators are required to compete to obtain sufficient research funding to run their laboratories, in addition to producing research results. In 2004, all Japanese national universities were incorporated as a National University Corporation under the National University Corporation Act (Japanese government, 2003). This change in policy has resulted in a reduction in national grant-in-aid funding and a rise in competitive funding. (Kikuchi, 2023). Principal investigators who are unable to obtain research funding are not able to conduct research, leading to a competitive, nerve-wracking environment.

Meanwhile, middle-senior researchers are generally required to produce research results within their fixed-term employment period (typically five years), and must also supervise students and secure research funding. Thus, the requirement to produce outstanding research results under unstable employment conditions places young researchers under immense pressure, which can lead to rushed research in closed laboratories, resulting in research misconduct. Principal investigators are also concerned about this situation, and are constantly negotiating with universities and research institutions in an effort to improve the employment environment. Enrolments in Japanese doctoral programs peaked in 2003, and have been on a downward trend since then (National Institute of Science and Technology Policy, 2022; Yonezawa, 2020). One reason might be the lack of employment opportunities for researchers coupled with the unstable job environment.

There are significant differences between the humanities and the natural sciences in terms of the evaluation and research processes. Previous studies have found that the natural sciences tend to involve group-based research activities, while the humanities tend to be more focused on individual research (Sørensen et al., 2021). It was noted that this makes it difficult to objectively prove new ideas and theories in the humanities. Evaluation of the relative achievements of researchers is not easy because the approach to measuring research performance differs between the humanities and the natural sciences.

Thus, it is important to consider how to evaluate researchers in various research fields. Our findings regarding the pressure on researchers to publish papers and the unstable employment conditions of junior researchers and middle-senior researchers supported those of PRINTEGER (Kennedy et al., 2018).

### Comparisons Between European and Japanese Interviews Based on the PRINTEGER Protocol

The results of the focus group interviews conducted as part of the PRINTEGER project were published in 2018 (Kennedy et al., 2018). The PRINTEGER interview protocol was translated and applied in this study. However, it was challenging to fully capture the original English-language nuances embedded in the questions. We acknowledge that cultural influences mediated through language might have affected participants’ interpretations. Moreover, comparisons between the present study’s findings and the original PRINTEGER findings must be approached with caution because the interviews were conducted at different times by different researchers. Despite these limitations, several similarities and differences were observed.

Regarding similarities, the concept of ‘good research’ was similarly understood in both regions as research conducted based on proper methodology. Likewise, ‘bad research’ was consistently associated with poor quality and methodology. Both groups also acknowledged the existence of a gray area between good and bad research, where boundaries remain ambiguous.

In terms of differences, Japanese participants tended to perceive research integrity as a singular, absolute concept, whereas European respondents demonstrated a more flexible and context-dependent understanding shaped by individual roles and experiences. With regard to research evaluation, Japanese participants primarily expressed the need for alternative indicators beyond publication quantity, while European respondents proposed concrete systems such as reward structures and incentives aligned with the promotion of research integrity. Finally, perceptions of the research community diverged: Japanese participants often viewed it as a closed and hierarchical environment, whereas European respondents described it as a collegial and constructive space for professional exchange.

### Limitations

First, for our focus group interviews, we recruited 25 graduate students, faculty members, and administrators from Kyoto University, with each of the five groups composed of just a few people, limiting the diversity of the data we obtained. In addition, some of the sessions were conducted during the COVID-19 pandemic, and the impact of the pandemic remains unknown. Japan is home to more than 800 universities, including national, public, and private institutions. Although Kyoto University, where this study was conducted, is one of the national universities, the findings presented herein do not necessarily reflect the perspectives of researchers affiliated with all Japanese universities. In future work, it would be worthwhile expanding the study to include other universities in Japan in an effort to obtain the opinions and ideas of many more researchers. Second, this study did not involve researchers who had either committed research fraud or had either witnessed or heard of fraud being committed, and thus the true underlying factors might not have been elicited. Third, there was a lack of detailed analysis of discipline-specific differences between the humanities and the natural sciences. Fourth, language is an important factor affecting ideas, cultural factors, and customs. In this study, the interviews and analysis were conducted in Japanese, and not all of the details were captured in the eventual English translation. Future studies should consider increasing both the range and number of participants, and broadening the scope of the questionnaires.

## Recommendations

As noted earlier, the findings of this study reflect the participants’ perceptions regarding the factors leading to research misconduct, rather than the actual causes. However, these perceptions suggest some measures that could be introduced in an effort to improve the research culture, including:

- Establishing a place where junior researchers, including postgraduate students from other research fields, can exchange information.
- Providing consultation for researchers regarding new regulations introduced in response to technological developments.
- Creating researcher-friendly guidance that aligns with the research environment.

## Conclusions

Comparisons among the focus groups revealed that despite the existence of factors that affect research integrity, people’s attitudes and behavior differ depending on their position and area of responsibility. Thus, improvements were suggested from the perspectives of the various positions. The creation of researcher-friendly guidance that is tailored to the research environment, the establishment of an environment facilitating interaction among junior researchers away from their laboratories, and the provision of consultation regarding new research regulations are recommended. We found that combining the knowledge of faculty, students, and administrators is the best way to foster a strong research culture and embed research integrity. Further research is needed to identify the factors that lead to research misconduct and how to address them.

## Electronic Supplementary Material

Below is the link to the electronic supplementary material.

Supplementary Material 1
