# Supplementary material for: Research Culture and Integrity in Japan: A Qualitative Study
Source: Sci Eng Ethics. 2026 Mar 14;32(2):20. doi: 10.1007/s11948-026-00591-2 (PMC13048944; doi:10.1007/s11948-026-00591-2)
Supplement: Supplementary file 1 — Supplementary Material 1 [file 11948_2026_591_MOESM1_ESM.docx]

Themes extracted from responses to Q1-1: Can anyone tell me why you conduct research, and what your motivations are? (Groups 1–3) (**Table 4**)

| Theme | Subtheme | Descriptive summary | Group |
| --- | --- | --- | --- |
| Pursuit of knowledge as a researcher | Interest in unresolved issues | - Interest and curiosity in being able to solve problems through research in a field in which I am interested | Group 1 (Junior researchers) |
|  | Contribution to the exploration of intellectual curiosity | - Contribution to the exploration of intellectual curiosity about one’s own research interests and to the development of medical science | Group 2 (Middle-senior researchers) |
|  | Contribution of research results to society | - Accomplishments in meeting new challenges and contributions of one’s own research results to others’ research | Group 3 (Senior researchers) |
|  | The research position as a profession - | - A research position as one of many occupations | Group 2 (Middle-senior researchers) |
|  | Occupation as a secondary choice | - Research position chosen as a result of the influence of others | Group 3 (Senior researchers) |

Themes extracted from responses to Q1-2: What makes good research? (Groups 1–3) (**Table 5**)

| Theme | Subtheme | Descriptive summary | Group |
| --- | --- | --- | --- |
| Scientifically sound research addressing societal challenges | Conducting research using the correct methodology | - Good research involves following the correct methodology based on an understanding of previous research, and addresses social problems and works toward solutions | Group 1 (Junior researchers) |

Themes extracted from responses to Q2: Defining bad research (Groups 1–5) (**Table 6**)

| Theme | Subtheme | Descriptive summary | Group |
| --- | --- | --- | --- |
| Poor quality research and inadequate methodology | Poor research practices | - Unanimous recognition of fabrication, falsification, and plagiarism as obvious research fraud, and concealment of bad research - Monopolization of research materials, self-interpretation of experimental data, lack of reproducibility of experiments, fabrication of research findings, inappropriate or excessive citation of previous studies, and exaggeration of research results | Group 1 (Junior researchers) |
|  | Plagiarism among students | - Reuse of reports among students and plagiarism via new technologies such as online information |  |
|  | Lack of qualifications and competence | - Questionable practices such as conveniently cited references, self-attribution of other people’s data, and lack of qualifications and competence as a researcher, in addition to fabrication, falsification, and plagiarism as research misconduct | Group 2 (Middle-senior researchers) |
|  | Inducing results for convenience | - Inducing results for your convenience or using statistically significant differences |  |
|  | Lack of responsibility for research | - Lack of responsibility for research data and lack of appropriate research attitude | Group 3 (Senior researchers) |
|  | Poor-quality papers | - Poor papers, including insufficient reference reading, conveniently cited references, and insufficient data to reach conclusions |  |
|  | Lack of research management | - Lack of comprehensive management skills, including recognition that research misconduct influences the entire organization | Group 3 (Senior researchers) |
|  | Issues in clinical research | - Concerns about statistical interpretation of data and arbitrary induction of results, and the applicability of study results to clinical practice | Group 2 (Middle-senior researchers) |
|  | Lack of consideration for subjects | - Lack of ethical consideration and poor study planning resulting in negative feelings among subjects | Group 3 (Senior researchers) |
| Pernicious authorship and citation | Customary authorship | - An established custom of inclusion of all members as authors in natural science laboratories | Group 1 (Junior researchers) |
|  | Coercion from journal referees | - Coercion from journal referees to cite their work during peer review |  |
|  | Questionable authorship | - Questionable authorship and plagiarism of research ideas among researchers | Group 4 (Research managers) |
| Dishonesty toward society and the research community | Sanctions against researchers | - Sanctions or expulsion from academic societies | Group 2 (Middle-senior researchers) |
|  | Distrust of society | - Distrust of science by the public | Group 3 (Senior researchers) |

Themes extracted from responses to Q3: Defining research integrity and what it means in practice for researchers (Groups 1–3) or for research governance advisors/managers (Groups 4 and 5) (**Table 7**)

| Theme | Subtheme | Descriptive summary | Group |
| --- | --- | --- | --- |
| Conceptual ambiguity and definitional gaps | Ambiguity as a result of the diverse range of research fields | - Boundary ambiguity regarding research integrity as a result of expansion and fusion among research areas | Group 1 (Junior researchers) |
|  | Borderline concerns | - Borderline concerns of misconduct and understanding the safe zone during research activities |  |
|  | Difficulties of interpretation as the opposite of research integrity | - Research integrity is a new term and concept, and there are difficulties regarding the definition as a result of interpretation, such as it being the opposite of research misconduct | Group 3 (Senior researchers) |
|  | Need for boundaries for research misconduct | - Clarification of the minimum rules that must be complied with, such as the boundary for misconduct, and the procedures that must be followed throughout the entire research process until it is completed | Group 4 (Research managers) |
|  | Definition of research integrity in every research area | - Varying perceptions of research integrity and research misconduct because of the current diversity in research fields and changes in research trends | Group 4 (Research managers) |
|  | Amendment of research integrity | - Amendment of research integrity as a result of changes in research trends, such as improved evaluation and increased social investment | Group 5 (Research administrators) |
| General recognition of research integrity | Adherence to research rules | - Adherence to rules, maintenance of objectivity and reproducibility, and prohibition of dishonesty | Group 2 (Middle-senior researchers) |
|  | General attitude as a researcher | - Attitude to publishing and sharing the results obtained following the implementation of shareable methods | Group 3 (Senior researchers) |
|  | Basic concept of research | - Research should be both a means of satisfying intellectual curiosity and accountable to society | Group 5 (Research administrators) |
|  | Qualities and attitudes as a researcher | - Researchers are educators regarding what is right and wrong who are typically sincere in their actions and results and are accountable to society for their own research |  |
| Relationship building between the public and researchers | Gap between society’s needs and research goals | - Gap between social needs and the researcher’s goals | Group 1 (Junior researchers) |
|  | Accountability to society | - Accountability to society regarding the significance of the research and its contribution to society | Group 2 (Middle-senior researchers) |
|  | Gap in thinking between society and researchers | - Gap in thinking between the public and researchers regarding the interpretation of research results | Group 3 (Senior researchers) |
|  | Expectations from society | - Expectations of excellent research results despite a limited research budget and a poor research environment | Group 5 (Research administrators) |

Themes extracted from responses to Q4: Research culture: expectations and management of research integrity (Groups 1–5) (**Table 8**)

| Theme | Subtheme | Descriptive summary | Group |
| --- | --- | --- | --- |
| Laboratory climate and educational context | Education through apprenticeship | - Distinction between educational guidance and harassment as a result of traditional apprenticeship practices in a laboratory | Group 1 (Junior researchers) |
|  | Issues related to the final decision | - Judgment of misconduct in the laboratory lacks any reference scale, and is based on the convenience of the group in an environment in which everything depends on the credibility of the laboratory leader |  |
|  | Environmental issues considered by junior researchers | - Patience of junior researchers in a laboratory environment dominated by old-fashioned supervisors - Some laboratory leaders have old-fashioned approaches to recognition and insight and cannot keep up with advances in analysis and analytical methods |  |
|  | Obstacles regarding researchers’ attitudes within the research community | - Bias regarding the number of research papers published has resulted in a lack of educational awareness and the harmful effects of a lack of education on research integrity in the laboratory |  |
|  | Research guidance methods in the laboratory | - Unconditional acceptance and secrecy regarding research methods within an isolated laboratory environment | Group 2 (Middle-senior researchers) |
|  | Closed laboratory management | - Closed laboratory management, resulting in limited or reduced information exchange with the outside world and lack of interaction among graduate students |  |
|  | Community rules in the laboratory | - Lack of awareness among principal investigators regarding research misconduct, limitations of self-monitoring, and the consequent functional breakdown of research groups |  |
|  | Obstacles that we cannot control ourselves | - Necessity of an environment for active discussion of research results and self-monitoring, for example, the existence of barriers between individuals or groups, such as small conflicts between academics, lack of communication, or lack of interest in other research fields |  |
|  | The creator and the user of the guidance are different | - Challenges of communication between those who provide research guidance and those who are guided, and the content of the guidance |  |
|  | Environment in which researchers feel cramped | - Inability to follow new research trends in the research environment | Group 3 (Senior researchers) |
|  | Rules and communication challenges in the lab | - Closed laboratory management, such as hierarchical relationships, and the harmful effects of blocking information and judgment in the laboratory |  |
|  | Environment around improper research | - An environment in which research misconduct can be detected at an early stage because of the development of new technology and tools | Group 4 (Research managers) |
|  | Background and factors leading to research misconduct | - Occurrence as a result of complex relationships among factors in the surrounding environment leading to research misconduct, and relationships between whistleblowers and non-whistleblowers | Group 5 (Research administrators) |
|  | Inadequate educational environment | - The need for research integrity education that fosters the qualities of researchers |  |
|  | Limitations on research administrators | - The limitations of unenforceable advice on research misconduct prevention |  |
| Positional issues of young researchers | Unstable position | - Researchers who have an unstable employment environment and a laboratory boss with absolute power | Group 1 (Junior researchers) |
|  | Vulnerable position of the younger generation | - Lack of study time for junior researchers during graduate school and demands from the research group that were beyond their ability at the time of writing their papers |  |
|  | Identity security and time-limited employment | - Existence of identity security and time-limited employment | Group 2 (Middle-senior researchers) |
|  | Barriers to conducting research for the younger generation | - High qualitative and quantitative demands on young researchers in terms of research achievements and the difficulty of mentoring students and enabling achievements by supervisory faculty | Group 3 (Senior researchers) |
|  | Evaluation of young researchers based on published papers | - A harmful environment for young researchers involving factors such as insecurity of their position because of evaluation based on the number of papers they have published | Group 4 (Research managers) |
| Challenges to conducting research for principal investigators | Challenges of running a laboratory | - Excessive competition for funding and positions, increase in information with the development of research equipment, and increase in non-research work, such as administrative and general tasks | Group 3 (Senior researchers) |
|  | Conflicts of interest faced by researchers | - Conducting research and promoting commercialization as part of the university’s mission |  |
|  | Conflicts of interest with external partners | - Efforts to obtain objective data and robust evidence - Conflicts of interest in relation to research funded by pharmaceutical companies |  |
|  | Differences in positions among organizations and individuals | - Differences in ideas among individuals and organizations, including differences in research objectives, such as researchers’ desire for research positions and contributions to society, and a closed environment with the Japanese artisan spirit or an open environment based on free discussion with researchers from other countries | Group 4 (Research managers) |
|  | Disparities between universities | - Disparities in the quality and quantity of faculty between large and small universities, and between mainstream faction and minority faction |  |
|  | External factors for researchers | - Maintaining organizational management skills, including performance during the years of career development, employment, competition for research funding, commitment to topics of strong social interest, and maintenance of research integrity | Group 5 (Research administrators) |
| Challenge facing reliability of research | Differences between the natural sciences and the humanities | - Differences in environment and characteristics between the natural sciences, featuring objective quantification based on experimental results and group research, and the humanities, featuring data collection and individual research - Areas of limited reproducibility of results in humanities research and areas of objective presentation based on data in natural science research | Group 2 (Middle-senior researchers) |
|  | Difficulty of interpretation in the humanities | - Difficulties based on previous research, such as the existence of differences in interpretation and understanding of the original text | Group 3 (Senior researchers) |
|  | Difficulty in publishing | - Difficulties in publishing new discoveries and theories in the humanities | Group 4 (Research managers) |
|  | Complexity of fraudulent decisions by the research field or organization | - Differences in the impact of research misconduct between the humanities and the life sciences | Group 5 (Research administrators) |
|  | Reliability assurance of research results | - Necessity for recognition of and trust in the quality of research from other research fields and disciplines | Group 2 (Middle-senior researchers) |
|  | Issues related to research involving human subjects | - Challenges of confidentiality and generalization of research related to personal information and difficulties in building relationships with families of subjects and other parties involved in the research | Group 4 (Research managers) |
|  | Limitations of research quality assurance | - Time limitations for students to complete their dissertations and the limitations of principal investigators’ guidance for students |  |
| Confusion over research rules | Flexibility to rule changes | Responding to changes in research culture and adjusting goals | Group 1 (Junior researchers) |
|  | Difficulties in research guidance in science areas | Enforcement of research rules for students, and difficulties for students in understanding experimental results and interpreting experimental results using new techniques | Group 2 (Middle-senior researchers) |
|  | Lack of understanding or questions about research integrity | Confusion regarding research ethics and the concept of research integrity, including research quality |  |
|  | Limitations of university policies | Challenges of academic policies without authorship arrangements | Group 3 (Senior researchers) |
|  | Increased burden of procedures | Excessive demands for compliance with rules and additional burdens | Group 4 (Research managers) |
|  | Policy transformation at the national level | A shift in national policy that disregards basic science |  |

Themes extracted from responses to Q5: Knowledge, education, and the impact of policies regarding research integrity on practice (Groups 1–5) (**Table 9**)

| Theme | Subtheme | Descriptive summary | Group |
| --- | --- | --- | --- |
| Policy limitations and the burden of learning research integrity | No effect on research despite lacking knowledge of research integrity | - Lack of knowledge or discussion about research integrity in a laboratory, with no effect on research | Group 1 (Junior researchers) |
|  | Differences in educational initiatives between other countries and Japan | - Practical education using foreign case studies and conceptual education using textbooks |  |
|  | Burden of acquiring research integrity | - Confusion as a result of numerous national and international guidelines and policies | Group 2 (Middle-senior researchers) |
|  | Burden of student education | - Time and effort for faculty to educate students on research integrity | Group 3 (Senior researchers) |
|  | Increased burden as a result of learning and enforcing research rules | - Difficulty keeping up with changes in rules and regulations, and the requirement to judge what is right and wrong |  |
|  | Limitations of policies to prevent misconduct | - Lack of understanding of policy content by researchers | Group 4 (Research managers) |
| Ethics education and researcher protection | Efficient guidance | - Flexibility of study item selection in the research field, avoidance of unnecessary content, and overall learning efficiency - Research guidance based on emphasis on the independence of individual researchers and the dangers of plagiarism in the humanities | Group 2 (Middle-senior researchers) |
|  | How to study the research rules | - Enhance learning methods using external research ethics education tools | Group 3 (Senior researchers) |
|  | Clarification of rules for education | - Clarification of rules to be followed, promotion of dissemination, and a student-first education system | Group 4 (Research managers) |
|  | System building for researcher protection | - Establishment of an environment in which researchers can discuss and eliminate factors related to research misconduct, and create organizational governance and safety nets, such as the appointment of an ombudsman | Group 5 (Research administrators) |

Themes extracted from responses to Q6: Support, interpretation, and translation of research integrity policies (Groups 1–5) (**Table 10**)

| Theme | Subtheme | Descriptive summary | Group |
| --- | --- | --- | --- |
| Enhancing ethics education and mitigating regulatory dissemination barriers | Overseas education for research integrity | - Conceptual education on social codes prior to the start of research, and practical learning opportunities through group work and lectures by external experts | Group 1 (Junior researchers) |
|  | Requests for a research integrity educational environment | - Determining the necessary concepts for the commencement of research and the scope of learning knowledge, and creating a comprehensive educational environment |  |
|  | Study period on research integrity | - Additions to the undergraduate student curriculum and the common university-wide curriculum | Group 2 (Middle-senior researchers) |
|  | Enhancement of research integrity educational environment | - Mandatory educational attendance and periodic revision of the educational environment | Group 4 (Research managers) |
|  | Creation of a system of regulations and ethics education materials | - Creation of a system of regulations based on the latest information and a mutual understanding by faculty and students based on the creation of ethics education materials |  |
|  | Confusion and trial-and-error when learning | - The complexity and difficulty of learning research integrity and teaching students as a result of a lack of experience regarding learning research integrity | Group 3 (Senior researchers) |
|  | Limitation of learning and use of punishment | - Limitations of widely recognized research integrity regulations and the use of severe punishment to shape research integrity | Group 5 (Research administrators) |

Themes extracted from responses to Q7: What works and what needs improvement? (Groups 1–5) (**Table 11**)

| Theme | Subtheme | Descriptive summary | Group |
| --- | --- | --- | --- |
| Performance evaluation | Need to create alternative methods of performance evaluation | - Creation of indicators and evaluation methods in addition to published papers for researcher evaluation | Group 1 (Junior researchers) |
|  | Measures that we can immediately implement ourselves | - Involvement of young researchers in creating mechanisms for building new systems | Group 2 (Middle-senior researchers) |
| Promoting transparency and integrity in research environments | Promoting research integrity | - Creation of an educational curriculum for basic knowledge and concepts of research integrity, and securing sufficient time to acquire them | Group 1 (Junior researchers) |
|  | Interaction among young researchers | - Interaction among young researchers across research fields, including the living environment for graduate students | Group 2 (Middle-senior researchers) |
|  | Measures that require cooperation from the university | - Learning methods that feel closer to research integrity, such as the use of group work and the granting of incentives, and the establishment of a consultation service within the organization |  |
|  | Fostering ethical consciousness | - Establishing ethics to be followed in research and fostering ethical behavior among researchers | Group 4 (Research managers) |
|  | Reinforcement of research review system | - Correcting generational differences between senior and young researchers, and improving the research review system to allow for open discussion |  |
|  | Curriculum development for research integrity | - Curriculum development for research integrity for undergraduate and graduate students, inter-departmental exchanges for faculty and staff, employment of full-time faculty for research integrity, and efficient use of e-learning | Group 5 (Research administrators) |
|  | Improvement in the preparation of papers | - Use of multilingual plagiarism software | Group 3 (Senior researchers) |
|  | Measures to prevent research misconduct | - Possibility of preventing research misconduct by improving relationships | Group 5 (Research administrators) |
| Promoting researcher well-being through supportive partnerships | Measures to actively encourage communication | - An environment that does not isolate young researchers and actively encourages communication | Group 3 (Senior researchers) |
|  | Measures to deal with the consultation system | - Establishment of a department for organizing the latest information and a consultation service regarding research integrity |  |
|  | Measures when conducting research | - Establishment of a department for organizing up-to-date information and a consultation service |  |
|  | Measures to provide guidance for students | - Student guidance based on the secondary advisor system among faculty members in other fields |  |
|  | New environment for research funding | - Provide and maintain an environment for obtaining new research funding, such as crowdfunding | Group 5 (Research administrators) |
|  | Building good relationships with research supporters | - Difficulty building relationships between research administrators and researchers and improving researchers’ awareness of the fact that their research is reliant on the support of numerous people |  |
| Sharing of past cases | Measures from case studies in the past | - Measures based on previous case studies, such as distancing from and self-defense against fraudulent research groups, severe punishment for those who engage in misconduct, and the need to establish a third-party committee | Group 2 (Middle-senior researchers) |
|  | Education using bad examples | - Education using bad examples of humanities research as teaching materials | Group 3 (Senior researchers) |
|  | Sharing post-disciplinary trends | - Sharing of disciplinary punishments for misconduct and reinstatement after disciplinary punishment | Group 4 (Research managers) |
|  | Sharing information on research misconduct cases | - Sharing of positive and negative examples after punishment | Group 5 (Research administrators) |
|  | Utilization as educational materials through the sharing of research misconduct cases | - Use of educational materials for prevention of recurrence, including prevention measures, improvement plans, and disciplinary punishments within the university |  |
